# Zebrafish sexual behavior: role of sex steroid hormones and prostaglandins

**DOI:** 10.1186/s12993-015-0068-6

**Published:** 2015-08-13

**Authors:** Ajay Pradhan, Per-Erik Olsson

**Affiliations:** Biology, The Life Science Center, School of Science and Technology, Örebro University, 701 82 Örebro, Sweden

**Keywords:** Brain transcriptomic, Forebrain, Midbrain, Hindbrain, Brain dimorphism, sexual behavior

## Abstract

**Background:**

Mating behavior differ between sexes and involves gonadal hormones and possibly sexually dimorphic gene expression in the brain. Sex steroids and prostaglandin E_2_ (PGE_2_) have been shown to regulate mammalian sexual behavior. The present study was aimed at determining whether exposure to sex steroids and prostaglandins could alter zebrafish sexual mating behavior.

**Methods:**

Mating behavior and successful spawning was recorded following exposure to 17β-estradiol (E2), 11-ketotestosterone (11-KT), prostaglandin D_2_ (PGD_2_) and PGE_2_ via the water. qRT-PCR was used to analyze transcript levels in the forebrain, midbrain, and hindbrain of male and female zebrafish and compared to animals exposed to E2 via the water.

**Results:**

Exposure of zebrafish to sex hormones resulted in alterations in behavior and spawning when male fish were exposed to E2 and female fish were exposed to 11-KT. Exposure to PGD_2,_ and PGE_2_ did not alter mating behavior or spawning success. Determination of gene expression patterns of selected genes from three brain regions using qRT-PCR analysis demonstrated that the three brain regions differed in gene expression pattern and that there were differences between the sexes. In addition, E2 exposure also resulted in altered gene transcription profiles of several genes.

**Conclusions:**

Exposure to sex hormones, but not prostaglandins altered mating behavior in zebrafish. The expression patterns of the studied genes indicate that there are large regional and gender-based differences in gene expression and that E2 treatment alter the gene expression pattern in all regions of the brain.

**Electronic supplementary material:**

The online version of this article (doi:10.1186/s12993-015-0068-6) contains supplementary material, which is available to authorized users.

## Background

Males and females exhibit characteristic biological differences that include phenotypical and physiological traits. Apart from gonadal differences, brain sexual dimorphisms have been demonstrated in mammals [1, 2]. Male and female brain differences affect biochemical processes, disease susceptibility, as well as behavior [1, 2]. The sex differences in behavior include courtship, mating, territorial marking, aggression, and parental care [2, 3]. The gonadal steroid hormones play a critical role in regulating sexual behavior [4]. Interestingly, while both 17-β estradiol (E2) and testosterone (T) elicit male sexual behaviors in mammals, the molecular mechanisms are poorly understood [4].

Sex steroids have been indicated to both activate and organize brain functions. Already during fetal development sex steroids permanently organize neuronal pathways involved in reproductive behavior and later, at puberty, sex steroids released form the gonads activate these differentiated pathways [5]. Through androgen receptor (AR) activation, T can both activate and organize neuronal pathways. T can also be converted to dihydrotestosterone (DHT) and E2 by steroid biosynthesis enzymes in the brain. E2 carries out its function via the estrogen receptors (ERα and ERβ) [3, 4]. Studies with mutant mice lacking AR have indicated that while male mice develop testicles and male secondary sexual characters they exhibit diminished male typical behavior [6, 7]. This has led to the formulation of a model where T and E2 have complementary roles in masculinization of the nervous system [6]. It has also been suggested that T aromatized to E2 amplifies male typical behavior while T acting through AR is needed to fully masculinize the brain [7].

T has been shown to masculinize the female brain in guinea pigs. Prenatal T treatment of female guinea pigs resulted in male typical behavior in adulthood, but the effect was not observed when T treatment was performed on females either perinatally or in adulthood [5]. This suggests that the brain is organized towards masculinization or feminization during the embryonic or fetal periods and that the gonadal hormones activate neural tissues for expression of male or female typical behavior in adult animals [5]. However, the presence of critical periods for gonadal hormone action in organizing or programing the nervous system remains controversial as it has been shown that sexual dimorphism of the brain can be repatterned postnatally [8]. As E2 is known to masculinize the female brain [9], T was assumed to be converted to E2 by aromatase to regulate brain patterning. Subsequent studies have shown that E2 is involved in induction of cyclooxygenase 2, a key enzyme in prostaglandin E_2_ (PGE_2_) synthesis which has been suggested to regulate sexual behavior in mice [10].

Gonadal hormones and prostaglandins can modulate the central nervous systems of birds and mammals, altering sexual behavior. However, there is insufficient data on teleosts to link hormonal roles to neural circuit organization and sexual behavior. Teleosts demonstrate a remarkable ability to undergo sex reversal both by hormonal treatment and socially controlled cues [11]. Bluebanded gobies live in groups consisting of a dominant male with several females and show sex reversal in both directions. Removal of the male from the group triggers the largest female within the group to undergo gonadal, morphological, and behavioral changes to become a fertile male. During this transition, aromatase activity drops quicker in the brain than in the gonads [11]. Fadrazole, an inhibitor of aromatase, and 11-ketotestosterone (11-KT) treatment of the blackeye goby resulted in protogynous sex change [12]. Interestingly, the administration of nonaromatizable androgens, 11-KT and 11-ketosandrosterone, resulted in sex change while the aromatizable 17α-methyltetosterone (MT) did not [12]. The gonadotropin releasing hormone (GnRH) neuron numbers in the preoptic area of the hypothalamus is higher in male bluehead wrasses and 11-KT treatment increases GnRH neurons and sex change in females [13]. When placed with a receptive female 11-KT treated female goldfish showed male typical behavior while T exposure resulted in low level of masculinization [14]. MT treatment has also been shown to induce male typical behavior in female goldfish [15] and in female three-spined stickleback [16]. On the other hand E2 treatment has been shown to result in male to female sex reversal in several different teleost species and affect male reproductive behavior [17–19]. This suggests that in teleost 11-KT induces male typical behavior in females and that E2 has feminizing properties.

Apart from gonadal hormones, prostaglandin is also known to regulate sex differentiation and sexual behavior in teleosts. Prostaglandin D_2_ (PGD_2_) and PGE_2_ regulate sex differentiation in zebrafish [20]. Inhibition of PGE_2_ synthesis by meloxicam, a non-steroidal anti-inflammatory drug (NSAID), resulted in an increased male to female sex ratio in zebrafish [20]. Ibuprofen, another NSAID, has been shown to decrease spawning in medaka [21]. In goldfish, prostaglandin (PGF_2α_) injections of males and females induced female typical spawning behavior in both males and females when paired with a control partner [15, 22]. PGF_2α_ and 15K-PGF_2α_ released from females act as pheromones to attract males [22]. PGF_2α_ and PGE_2_ can also down-regulate the gonadotropin levels in goldfish [23]. Taken together this suggests that prostaglandins can have a significant effect on teleost gonadal and brain functions.

While hormone [24, 25] and prostaglandin [20] treatment of zebrafish has been shown to result in sex reversal less is known about the role of gonadal hormones in brain organization and activation of sexual behavior. However, transcriptomic studies have shown that the zebrafish brain is sexually dimorphic [26–28]. Compared to the adult mammalian brain the adult zebrafish brain shows a higher potential for neurogenesis [29] suggesting that the zebrafish brain is more plastic and that chemical or hormonal treatment could induce activational and/or organizational effects, thus altering sexual behavior. To explore the activation of sexual behavior zebrafish were exposed to sex steroids and prostaglandins. Since gonadal hormones and prostaglandins can cause sex reversal in zebrafish, the exposure was performed on adult fish to determine if sexual behavior could be altered during the active reproductive stage.

## Methods

### Zebrafish maintenance and exposure

Wild type zebrafish were obtained from a local pet store in Sweden. The experiments were performed using 6–8 months old zebrafish of F2 and subsequent generations were maintained in a recirculating system (Aquaneering, USA) with a 14 h light/10 h dark cycle at 26–27°C. The fish were fed twice a day with flake food (Tetrarubin, Germany) and *Artemia* nauplii (Ocean Nutrition, Belgium). The adult zebrafish were exposed individually to 11-KT, E2, PGE_2_ and the PGD_2_ analog BW 245C (Sigma, USA). All compounds were dissolved in water and added to 2 L exposure glass beakers to 1 L final volume. Due to the short half-life of prostaglandins, water was completely changed each day. Steroid hormone water was changed every second day. The permits for the animal experiments were obtained from the Ethical Committee in Linköping, Sweden (Permit 32-10).

### Brain tissue sampling

Adult male and female zebrafish were exposed to 25 nM E2 for 24 h. The brains were then removed and dissected into three regions: (1) forebrain containing olfactory bulb and telencephalon; (2) midbrain containing tectum, hypothalamus and pituitary; (3) hindbrain containing cerebellum and medulla. The tissue samples were snap frozen in liquid nitrogen and stored at −80°C until further use.

### Sexual behavior

Zebrafish used for behavioral experiments were maintained in individual 2 L aquaria. In order to select for sexually active fishes, every 10 days one male and one female zebrafish were placed together and allowed to spawn. For each experiment six couples were used and the experiment was repeated 4 times. After spawning successfully 3–4 times, the male or female zebrafish were individually exposed to E2 (25 nM), KT (25 nM), PGE_2_ (20, 50 and 100 nM) or BW 245C (20, 50 and 100 nM) for 8 days. On day 9 the treated fish was placed with its control partner. The following morning sexual behavior was observed (male chasing, female being chased and egg release by the females) for 30–40 min. This time was chosen as zebrafish are sexual active immediately after illumination [30]. Male and female mating behavior and spawning was recorded.

In a separate experiment six female zebrafish were individually exposed to E2 (25 nM), 11-KT (25 nM), PGE_2_ (20, 50 and 100 nM) or BW 245C (20, 50 and 100 nM) and paired with a control female. The tail fin of the exposed female was cut to identify it from the control female. For E2 and 11-KT, the experiment was performed four times (n = 6, 4 repetitions) and for prostaglandins the experiment was performed twice (n = 6, 2 repetitions) with different batches of fish. After exposure termination, the fish were kept in normal water and paired again after 2 weeks to observe if the behavioral changes were temporary or permanent. On completion of the behavior studies, the fish were euthanized and sex was determined by gonadal morphology.

### RNA extraction and quantitative RT-PCR

RNA extraction was performed using the NucleoSpin RNA II kit (Macherey–Nagel, Germany) and qRT-PCR analysis was performed as described previously [31]. RNA was quantified using the NanoVue (GE Healthcare, UK) and equal amounts of RNA (500 ng) was used for cDNA synthesis using the qScript cDNA synthesis kit (Quanta Biosciences, USA). Primers were designed for genes that are involved in stress response, sex differentiation, and regulation of brain functions (Additional file 1: Table S1). SYBR Green (Kapa Biosystems, USA) with 150 nM each of forward and reverse primers was used to determine the expression levels of all genes. Thermocycling conditions for SYBR Green consisted of a denaturation step for 5 min at 95°C followed by 40 cycles of 95°C for 2 s and 60°C for 30 s. Four biological and two technical replicates were used to study gene expression and the transcript levels were normalized using a housekeeping gene, *elongation factor 1a1* (*ef1a1*). To avoid use of cross plate reference, each gene was run in one plate. Heat map for analyzed transcript levels were generated using Tableau software, version 8.2 (Tableau software, USA).

### Statistical analysis

The Mann–Whitney U*-*test and Kruskal–Wallis test followed by Dunn’s multiple comparison test was used to analyze behavioral data. For the behavioral studies, the untreated couples (male–female or female–female couples) were used as controls. The differences were considered statistically significant if the p value was <0.05 (*).

Statistical analysis of the qRT-PCR data was performed using the two-tailed non-paired Student’s t-test for two group comparison and one way analysis of variance (ANOVA) followed by Dunnett post-test for multiple group comparison. The false discovery rate (q < 0.05) was determined using the FDR online calculator (http://www.sdmproject.com/software/) and used to adjust the p values. To further determine if the parametric statistical analyses agreed with non-parametric statistical analyses Kruskal–Wallis test followed by Dunn’s multiple comparison as well as Mann–Whitney U-test was used to confirm or reject the results from the parametric analysis. Statistically significant differences that remained after these tests were considered significant if the P values (parametric tests) were <0.05 (*p < 0.05; **p < 0.01; ***p < 0.001). Statistical analyses were performed using the GraphPad Prism 5 software (GraphPad Software).

Principal component analysis (PCA) is a multivariate data analysis method that incorporates all the data and variables to determine the principal components contributing to the correlation patterns of the data by giving low preference to variables that show non-significant variance and identifying variables and data showing significant variances. The multivariate data analysis and PCA were performed using the SIMCA software, version 13.0.3 (Umetrics, Sweden) at a significance level of 0.05. A score plot showing sample grouping and a loading plot indicating the relationship between the variables was used in the analysis. Values that explain the variation, R^2^X > 0.7 (goodness of fit) and Q^2^ > 0.4 (goodness of prediction) were considered to denote an acceptable model when analyzing biological data.

## Results and discussion

### Mating behavior and spawning

Sex specific behavior including mating behavior, aggression and territorial marking is very common among sexually reproducing animals. During development the male and female brain become differentially organized and both steroid hormones and prostaglandins can activate and alter sexual behavior in mammals as well as in teleosts. In the present study we aimed to determine the influence of exogenously administered sex hormones and prostaglandins on zebrafish mating behavior, spawning and fecundity. Furthermore, analysis of gene expression of male and female zebrafish brains, as well as the effect of E2 exposure was performed to identify sex dependent differences in gene expression.

**Figure 1:**
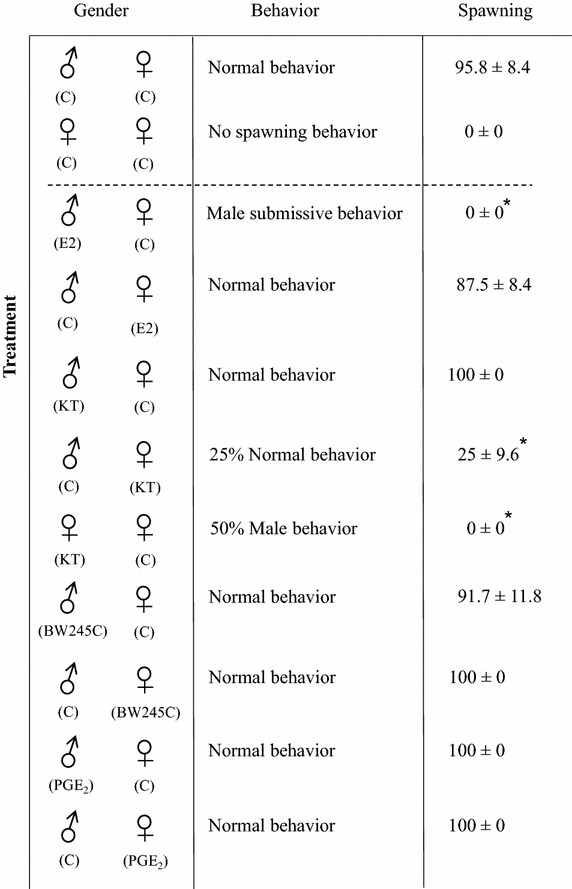
Behavioral change and spawning. Male and female zebrafish were exposed to 25 nM of sex steroids (E2 and 11-KT) and 20, 50 and 100 nM of prostaglandins (PGE_2_ and BW245C) separately for 8 days then kept together to analyze any change in behavior. Kruskal–Wallis test followed by Dunn’s post-test was performed to determine any change in normal behavior between the control and treated groups (*p < 0.05). Mann–Whitney U-test was used to determine statistically significant differences between control and KT exposed females paired to untreated females (*p < 0.05). The steroid hormones experiments were performed with 24 fish while 12 fish were used for prostaglandin exposures.

There were significant changes in mating behavior and spawning success following exposure to E2 and 11-KT (Figure 1). Male fish exposed to 25 nM E2 showed no male mating behavior and no spawning was observed in this group. Earlier studies on zebrafish have shown that exposure to 17α-ethynylestradiol (EE2) result in female-biased sex ratios, and that males that did not undergo sex reversal showed either unaltered male sexual behavior [32] or reduced sexual behavior [33, 34]. In another study EE2 exposed zebrafish males that did not undergo complete sex-reversal but that had reduced testis size or rudimentary ovaries were able to induce spawning behavior when placed with control female [35]. In a separate study it was shown that exposure of zebrafish to low levels of EE2 had no effect on male behavior but rendered the females less interest in EE2 exposed males [36]. Exposure to E2 has been shown to result in decreased male reproductive behavior in other teleosts as well [17–19].

Exposure of female zebrafish to 25 nM 11-KT resulted in reduced female mating behavior with only 25% showing normal mating behavior and spawning (Figure 1). Fifty percent of the female fish exposed to 25 nM 11-KT and paired with a control female showed male mating behavior but did not induce spawning in the control female. 11-KT and MT have been reported to induce male typical behavior in female goldfish [14, 15]. This suggests that androgen exposure in teleost leads to masculinization of the female brain. In this study we did not observe spawning (egg release) from control females when paired with 11-KT exposed females. However, as spawning behavior is very similar to aggressive behavior we cannot differentiate between mating behavior and aggressive behavior using the present setup.

Since, PGE_2_ and PGD_2_ are known to influence sex differentiation in zebrafish [20] and as PGE_2_ is involved in masculinization of female mice [10], we included these two prostaglandins to determine whether they could alter sexual behavior. However, treatment with three different concentrations (20, 50 and 100 nM) of PGE_2_ and BW245C did not result in any apparent change in sexual behavior or spawning (Figure 1). Injection of PGE_2_ and PGF_2α_ in the third ventricle of goldfish has been shown to down-regulate gonadotropin [23] which suggests that prostaglandins could alter sexual behavior in teleosts. Spawning was induced in PGF_2α_ injected female goldfish while injected males showed female typical behavior [15]. PGF_2α_ along with 15K-PGF_2α_ released from female goldfish can act as pheromones inducing male typical behavior and synchronizing sperm release with egg release [22, 37, 38]. Due to the short half-life of PGF_2α_, the preferred method of exposure has been through injection, with high efficacies when injected into the ventricles of the brain [38]. The roles of prostaglandins in zebrafish sexual behavior clearly require additional studies.

### Brain region differential gene expression

Zebrafish whole brain transcriptomic analyses have identified sexually dimorphic gene expression patterns [26–28]. In order to achieve better resolution we dissected the brain into three regions. This allowed us to determine region-specific expression patterns and to identify sex differences in gene expression. In addition we exposed zebrafish to E2 to determine its role in region specific alteration in gene expression and steroid hormone mediated alterations in sexual behavior. A set of 32 genes was selected for analysis. The selection was based on their involvement in brain functions, sex differentiation and stress responses.

**Figure 2:**
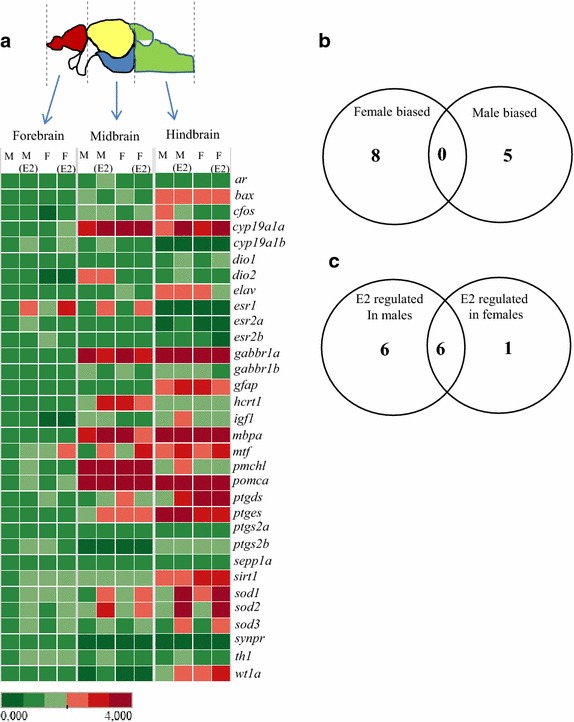
qRT-PCR array. Male and female zebrafish (n = 4 for each sex) were exposed separately to 25 nM E2 for 24 h and the brain tissue was isolated and dissected into three regions. RNA was isolated from individual samples followed by qRT-PCR analysis of 32 genes. **a** Tableau software was used to generate the heat map. The scale bar represents magnitude of expression with male sample from forebrain set to 1 and highest expression fixed to 4. **b** Venn-diagram showing the sexually dimorphic genes with higher expression of 8 genes in females while only 5 genes were male biased. 19 genes did not show sexually dimorphic expression patterns. **c** Venn-diagram showing E2 mediated dimorphic gene expression with six genes being regulated in males only, six genes being regulated in both sexes and one gene being regulated in females only.

The three brain regions showed distinct differences in genes expression levels with an overall higher expression in the mid- and hindbrain regions compared to the forebrain (Figure 2a). The *baxa*, *elav*, *gfap*, *gabbr1a*, *mbpa*, *mtf1*, *ptgds*, *ptges*, *sirt1* and *wt1a* genes showed the highest expression in the hindbrain region (Additional file 2: Figure S1–3). Of these genes the *gabbr1a*, *mbpa*, *mtf1*, *ptges* and *sirt1* genes showed intermediate expression in the midbrain and the lowest expression levels in the forebrain. Three genes (*dio2*, *pmchl* and *pomca*) were highly expressed in the midbrain region (Additional file 2: Figure S1–2). The dio2 gene that was also highly expressed in the hindbrain region is involved in regulation of thyroid hormone levels. The *Pro*-*melanin concentrating hormone*-*like protein* (*pmchl*) gene and *proopiomelanocortin a* (*pomca*) gene are involved in melanin regulation. Both Pomca and Pmchl are also neuroactive peptides, synthesized in the hypothalamus and suggested to control appetite and energy homeostasis [39, 40]. The expression levels of pomca and pmchl were 77 fold and 95 fold higher in the midbrain than in the forebrain, which could be due to high expression of these genes in the pineal gland present in the midbrain. These two genes showed the highest region specific variation of the tested genes. Only one gene, *synaptoporin* (*synpr*), showed the highest expression in the forebrain. The *cyp19a1b*, *esr1* and *esr2a* genes showed the lowest expression in the hindbrain (Additional file 2: Figure S1). *Cyp19a1*b is the brain form of aromatase and it was interesting to note that this gene was not up-regulated by estrogens in the hindbrain region. Overall the hindbrain region showed the largest differences from the other two regions, with ten highly expressed genes and three genes with low expression. As the cerebellum is involved in motor control and as it showed the lowest levels of cyp19a1b, esr1 and esr2a estrogen regulated genes it was of interest to determine sex differences in this region and how estrogen treatment would affect this brain region.

### Sex biased gene expression

Out of 32 genes analyzed, 13 genes showed sexually dimorphic expression with 8 genes being female biased and 5 male biased (Figure 2b; Table 1). Female-biased genes expression was observed for two *estrogen receptors* (*esr1 and esr2b*), *metal transcription factor 1* (*mtf1*), *prostaglandin synthase 2b* (*ptgs2b*), *sirtuin 1* (*sirt1*) and *superoxide dismutase 1* (*sod1*) in the forebrain region (Additional file 2: Figure S1–3). The genes for *cytochrome P450*, *family 19*, *subfamily A*, *polypeptide 1b* (*cyp19a1b*), and the PGD_2_ synthesis enzyme *prostaglandin D synthase* (*ptgds*) were high in the hindbrain region (Table 1). *Ptgds* expression has been observed to be lower in perinatal males mice than in females [41] and has been suggested to be involved in neuroprotection [42]. Ptgds is involved in testis differentiation in mammals [43] and zebrafish [20] but there is no data to link differential expression in the brain with that of sexual behavior.

**Table 1:** Genes showing significant dimorphic expression in the three brain regions

Genes	Male/female^a^ biased	p-value*		
		Forebrain	Midbrain	Hindbrain
*c*-*fos*	Male	ns	0.0087	ns
*cyp19a1b*	Female	ns	ns	0.0090
*dio2*	Male	0.0017	0.0004	0.0256
*esr1*	Female	0.027	ns	ns
*esr2b*	Female	0.0166	ns	ns
*gabbr1a*	Male	ns	ns	0.0211
*gabbr1b*	Male	ns	ns	0.0185
*igf1*	Male	<0.0001	ns	ns
*mtf*	Female	0.0045	ns	ns
*ptgds*	Female	ns	ns	0.0007
*ptgs2b*	Female	0.0235	ns	ns
*sirt1*	Female	0.0007	ns	ns
*sod1*	Female	0.0121	ns	ns

*ns* not significant.

^a^Females showed higher expression of eight genes and males showed higher expression of five genes.

* Student’s t test was performed to determine statistical significance.

Male biased gene expression was observed for *insulin*-*like growth factor 1* (*igf1*) in the forebrain region. *v*-*fos FBJ murine osteosarcoma viral oncogene homolog* (*c*-*fos*) was higher in the midbrain region and *gamma*-*aminobutyric acid B receptor 1a and 1b* (*gabbr1a and gabbr1b*) were higher in the hindbrain region (Table 1). The expression of the *deiodinase*, *iodothyronine*, *type II* (*dio2*) gene was higher in males than in females in both the fore- and midbrain regions (Table 1).

GnRH neurons are higher in male wrasse and induction of sex change by 11-KT in female teleosts has been implicated to increase the number of GnRH neurons [13]. Igf1 has been shown to induce GnRH in salmon and zebrafish [44, 45] and Igf1 is also involved in the organization of GnRH neurons by influencing migration and differentiation of neural crest cells in juvenile zebrafish [45]. The male-biased *igf1* expression in zebrafish forebrain (Additional file 2: Figure S2D) suggests that *igf1* could be involved in male behavioral patterning in teleosts. GnRH has been shown to induce c-Fos in mammals, leading to up-regulation of the gonadotropin genes [46]. In the present study *c*-*fos* expression was higher in the male forebrain and midbrain regions (Additional file 2: Figure S1C) strongly correlating with the *igf1* expression in the forebrain. GnRH signaling together with Igf1and c-Fos may thus be involved in zebrafish sexual behavior.

In the present study expression of *esr1* was higher in the fore- and midbrain than in the hindbrain (Additional file 2: Figure S1H). ER expression and function can vary depending on the region of expression. ERα has been suggested to control reproductive neuroendocrine functions while ERβ in regulate non-reproductive functions, including anxiety related behavior [47]. ERβ has also been linked to the defeminization of the mammalian male brain [48], reduced sexual activity, and sterility in both male and female mice [49, 50]. Brain aromatase (*cyp19a1b*), that is involved in E2 synthesis in the brain, showed higher expression in the hindbrain of females (Additional file 2: Figure S1). Exposure to the aromatase inhibitor fadrazole caused protogynous sex change in the blackeye goby by reducing the E2 levels [12]. Fadrazole treatment also induced female to male sex change in zebrafish [51]. In male rats fadrazole leads to reduction in ejaculation and intromission [52], which suggests that E2 is important for masculinization of the mammalian brain while it feminizes the teleost brain.

### 17β-estradiol induced gene expression

E2 treatment resulted in up-regulation of ten genes and down-regulation of three genes in different brain regions (Figure 2c). The *esr1* gene was up-regulated by E2 in all brain regions of both sexes. Three genes, *mtf1*, *sod1* and *sod2* were up-regulated in all brain regions in male fish. The *sod* genes showed region specific expression in females with s*od1* being up-regulated in the hindbrain, *sod2* being up-regulated in both the mid- and hindbrain and *sod3* being up-regulated in the fore- and midbrain. Three genes were down-regulated by E2 and these included *esr2b* (down-regulation in female fore- and midbrain), *gabbr1a* (male hindbrain) and gbbr1b (male mid- and hindbrain). It is interesting that the hindbrain region, that showed low expression of *cyp19a1b*, *esr1* and *esr2a*, also showed minor up-regulation of *esr1* and no regulation of *esr2a* or *cyp19a1b* in female hindbrain following E2 treatment. In order to discriminate between the roles of different ER isoforms it should be possible to use ER specific inhibitors, such as propyl-pyrazole-triol for ERα and diarylpropionitrile for ERβ separate their roles in modulating teleost sexual behavior. In male zebrafish E2 up-regulated *cyp19a1b* (fore- and midbrain) and *esr2a* (hindbrain), suggesting a sex specific E2 regulatory pattern in this brain region. In a study on mice *AR* showed higher expression in males than in females and while T down-regulated *AR* in both sexes E2 only up-regulated *AR* in male brains [53]. In the present study we did not observe sexually dimorphic expression of zebrafish *ar.* It was interesting to note that *tyrosine hydroxylase* (*th1*), the rate-limiting step in catecholamine synthesis including dopamine (DA), was up-regulated in male forebrain by E2. DA is involved in modulation of aggressive behavior in zebrafish [54] and in teleost DA is known to inhibit reproduction [55]. The E2 mediated up-regulation of *th1* in this study indicates that DA level could be involved in alteration of sexual behavior in treated males.

### Multivariate data analysis

**Figure 3:**
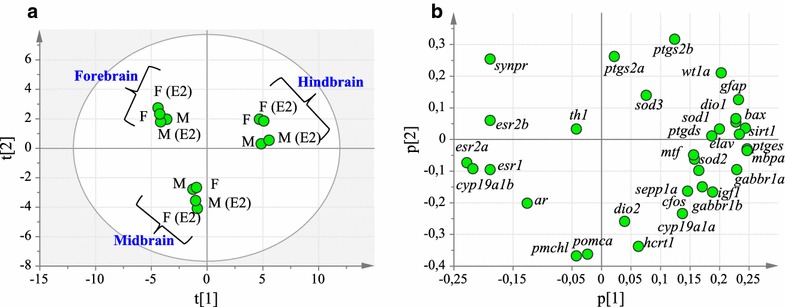
Principal component analysis. The PCA analysis was based on the gene expression patterns in male (M) and female (F) zebrafish brain regions with and without E2 exposure. Gene expression in the forebrain, midbrain and hindbrain regions were analyzed. **a** Score plot showing the distribution of the brain regions in control and E2 exposed zebrafish based on gene expression. The ellipse shows Hotellings T^2^ (0.05). **b** PCA loading plot showing the distribution of the measured gene expression levels.

Principal component analysis (PCA) was used to analyze the correlation between gene expression levels and brain regions (Figure 3). The PCA plot comparing the three brain regions with and without E2 exposure gave a model (R^2^X = 0.839 and Q^2^ = 0.561, three component model) showing that the three brain regions had distinct gene expression patterns. PC1 explained 49.5% of the variation while PC2 and PC3 explained 20.4 and 14.0% respectively. The PCA score plot showed that male and female control groups as well as treated groups cluster together according to brain region (Figure 3a). The PCA loading plot showed that the expression of majority of the genes was higher in the hindbrain region than the two other regions (Figure 3b). The data shows that the expression of genes involved in androgen and estrogen regulation (*ar*, *esr1*, *esr2a*, *esr2b* and *cyp19a1b*) correlated to localization to the forebrain and midbrain regions. Analysis of the effect of E2 treatment on gene expression did not show any significant differences using the PCA analysis. The R^2^X value was below 0.7 when comparing the gene expression between male control and male E2 in the forebrain. All other comparisons gave R^2^X values above 0.7. However, the Q^2^ values were below 0.4 indicating that the data cannot be used to predict the expression patterns. Likewise, comparisons of gene expression between male and female brain regions gave R^2^X values >0.7 but Q^2^ values below 0.4. Increasing the number of animals and the number of genes could be of interest in order to better identify overall differences between the brain regions and hormone treatment. The present data suggest that the overall gene expression patterns are highly similar between sexes and following hormone treatment. However, the data do indicate that there are large differences between brain regions, independent of sex.

## Conclusions

This study shows that hormonal treatment has an activational role in the zebrafish brain and that the effect of prostaglandins, though not observed in this study cannot be ruled out. E2 treatment appears to feminize the male brain while 11-KT masculinizes the female brain. The ability to induce male and female typical behavior by steroids in opposite sex, suggests that zebrafish have sexually bipotential brains that can be primed either to male or female sexual behavior. Further studies with hormonal exposure and analysis of neuronal activity at both transcriptional and protein level will help to better understand the mechanisms of hormonal action on the teleost brain. This study provides useful information and possible future directions to help unravel the long unsolved mechanisms of gonadal hormones in masculinization as well as feminization of neonatal brain.

## Additional files

Additional file 1:
**Table S1.** Primers used for qRT-PCR analysis. Additional file 2:
**Figure S1.** Male and female zebrafish were exposed separately to 25 nM E2 for 24 h and the brain tissue was isolated and dissected into three regions. RNA was isolated from individual samples followed by cDNA synthesis and qRT-PCR analysis. Statistical significance was determined as outlined in the materials and methods section. Statistically significant differences were determined using Student’s t test (*p < 0.05; **p < 0.01; *** p < 0.001). n = 4. **Figure S2.** Male and female zebrafish were exposed separately to 25 nM E2 for 24 h and the brain tissue was isolated followed by cDNA synthesis and qRT-PCR analysis. Statistical significance was determined as outlined in the materials and methods section. Statistically significant differences were determined using Student’s t test (*p < 0.05; *** p < 0.001). n = 4. **Figure S3.** Male and female zebrafish were exposed separately to 25 nM E2 for 24 h and the brain tissue was isolated followed by cDNA synthesis and qRT-PCR analysis. Statistical significance was determined as outlined in the materials and methods section. Statistically significant differences were determined using Student’s t test (*p < 0.05; **p < 0.01; *** p < 0.001). n = 4.
