# Supplementary material for: Zebrafish sexual behavior: role of sex steroid hormones and prostaglandins
Source: Behav Brain Funct. 2015 Aug 13;11:23. doi: 10.1186/s12993-015-0068-6 (PMC4575480; doi:10.1186/s12993-015-0068-6)
Supplement: Additional file 1: — Table S1. Primers used for qRT-PCR analysis. [file 12993_2015_68_MOESM1_ESM.docx]

**Supplementary Table.**

Table S1. Primers used for qRT-PCR analysis

|  |  |  |
| --- | --- | --- |
| ***Gene symbol*** | **5' primer** | **3' primer** |
| *ef1a1/1* | GTACTACTCTTCTTGATGCCC | GTACAGTTCCAATACCTCCA |
| *ar* | ACAACACACCTGGATGGGAGTGAT | TGACCTGTAGCAGCACAAACTCCT |
| *baxa* | CGCCTCAACCCAATGAAAGAGGA | ACAAGAAAGGGCACAGACTCTGGA |
| *Cyp19a1a* | GGAGACATTGTGCGTGTCTGGA | AGAATGTCCAGCTGTCCTTGAGCA |
| *Cyp19a1b* | CATGCAGCCAGTAGAAGAGGA | TGAGCTCGCGGGATGA |
| *cfos* | TACACTGCAAGCTGAAACTGACCA | TTGTGTGCGGCGAGGATGAA |
| *dio1* | GTTCAAACAGCTTGTCAAGGACT | AGCAAGCCTCTCCTCCAAGTT |
| *dio2* | GCATAGGCAGTCGCTCATTT | TGTGGTCTCTCATCCAACCA |
| *elav* | AGACAAGATCACAGGCCAGAGCTT | TGGTCTGCAGTTTGAGACCGTTGA |
| *esr1* | AAGAACTCGTCCACATGATCGCCT | AGACTCCGAAATCGAGCCACAGTT |
| *esr2a* | TACGACTTCAGCACTCTGCCCTTT | CCGCTTTACCAGTGGTTTGCTGTT |
| *esr2b* | TGTTCGAGTTTGCCACAGACTCCT | ACAGATGCTCGATGCCTTTGTTGC |
| *gabbr1a* | CCCAGAGACGGAGGGATACG | CGGGCACATCATCAAGCATCT |
| *gabbr1b* | AGGTGTTGCCGGTGGATTATGAAA | TGGAGGATGAACCGTCACACG |
| *gfap* | TTTCCTGAAGAAGGTCCATGA | GACACATCCAGATCCACATGA |
| *hcrt1* | GGCTCCTGCAAACTCTACGA | GAATTTAGCGGGCTCCTCCA |
| *igf1* | TGAGATGTGACATTGCCCGC | AGCGCATGGTACACTTAAAGACA |
| *mbpa* | AATCAGCAGGTTCTTCGGAGGAGA | AAGAAATGCACGACAGGGTTGACG |
| *mtf1* | GGTGAAGAGATACCAGTGTCTGTTCGA | TGGTGAAGTACTTGGTGCATCCCT |
| *pmchl* | ACCGCTAAAGCAAACGCTCA | CTGGAGCCACCAGGTGTAGA |
| *pomca* | CCTCAGCACAGAGGAGAACA | CGGGGGTTTCATCTGTAAGT |
| *ptgds* | CGGGTGGTTGACGCCAAGT | CGGCAACTGAGCATTCTCCA |
| *ptges* | GAGCTGCTGCCATGTGGATC | CATGTTGTGTGGCCTTCTGCA |
| *ptgs2a* | CACTGTTGCCGGACAACTTTCAGA | TCCAGCAGTCTGTTTGGTGAAGGA |
| *ptgs2b* | CTTTACCATTGGCACCCCCT | CCACCCTTAACACTGCTGGT |
| *sirt1* | CAGCTCTGCTACAATTCATCGCGTC | AATCTCTGTAGAGTCCAGCGCGTGTG |
| *sepp1a* | ATCCTGAGCCACCCACAT | ACTCTTCAAGTTGCGCTGAA |
| *sod1* | TGAGACACGTCGGAGACC | TGCCGATCACTCCACAGG |
| *sod2* | TTCAGGGCTCAGGCTGG | ATGGCTTTAACATAGTCCGGT |
| *sod3* | GCCTCGTGTGTATGGTCACA | TGGATGGTTGACGTTGAGGG |
| *synpr* | ACCCATCTGGACCAATCTCA | AGGGATACCGCTGGAAAGAC |
| *th1* | TTGATCGAGGATGCGCGTAA | TTTGGTTTTCGGCTGGGTCT |
| *wt1a* | GAGCCATCCCGGAGGTTATGA | TTGGTCTCGGTTGAACGCACA |
|  |  |  |
