# Supplementary figures and images for: Zebrafish sexual behavior: role of sex steroid hormones and prostaglandins
Source: Behav Brain Funct. 2015 Aug 13;11:23. doi: 10.1186/s12993-015-0068-6 (PMC4575480; doi:10.1186/s12993-015-0068-6)

## Slide 1
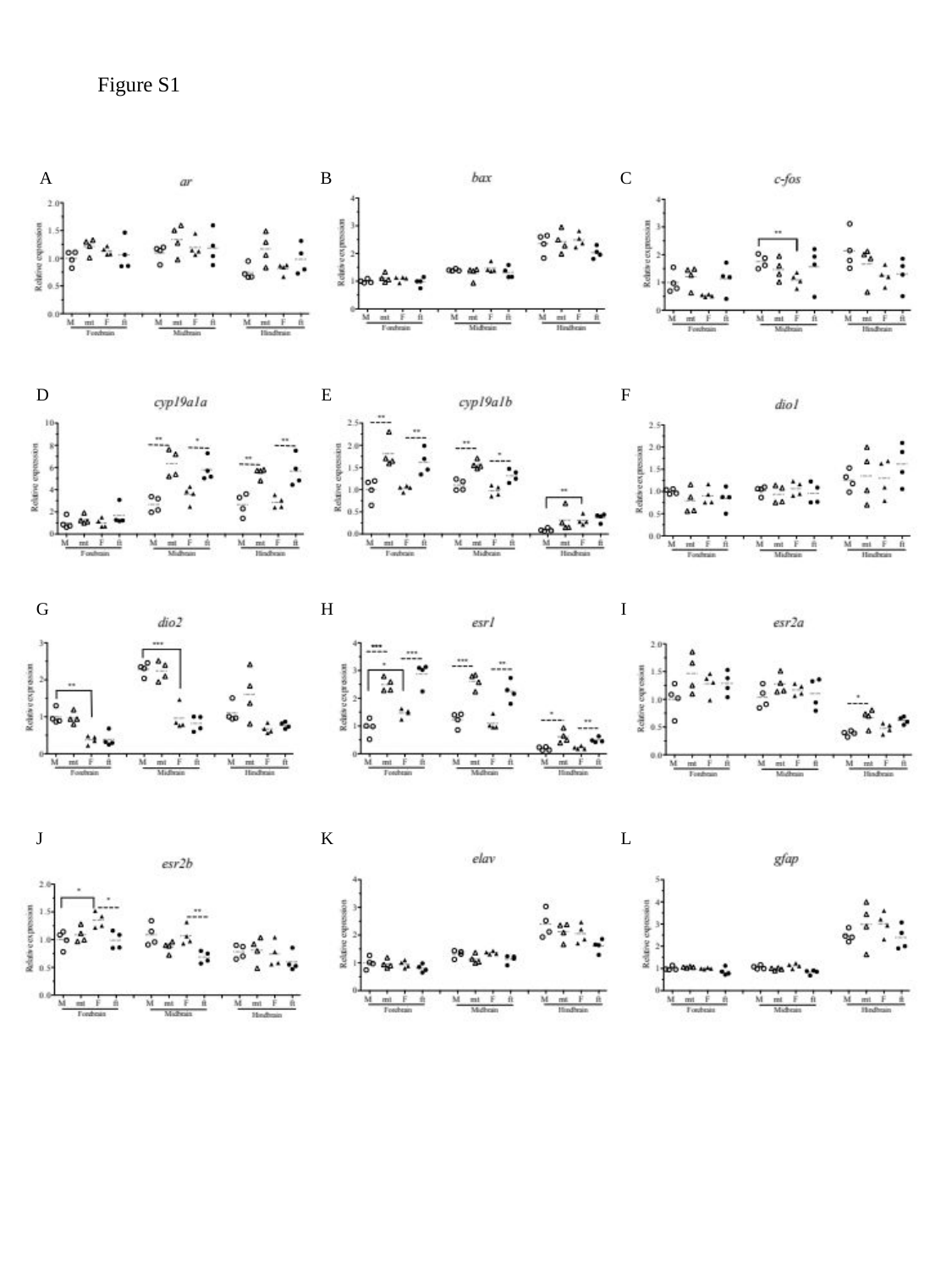

Figure S1
B
A
C
E
D
F
H
G
I
K
J
L

## Slide 2
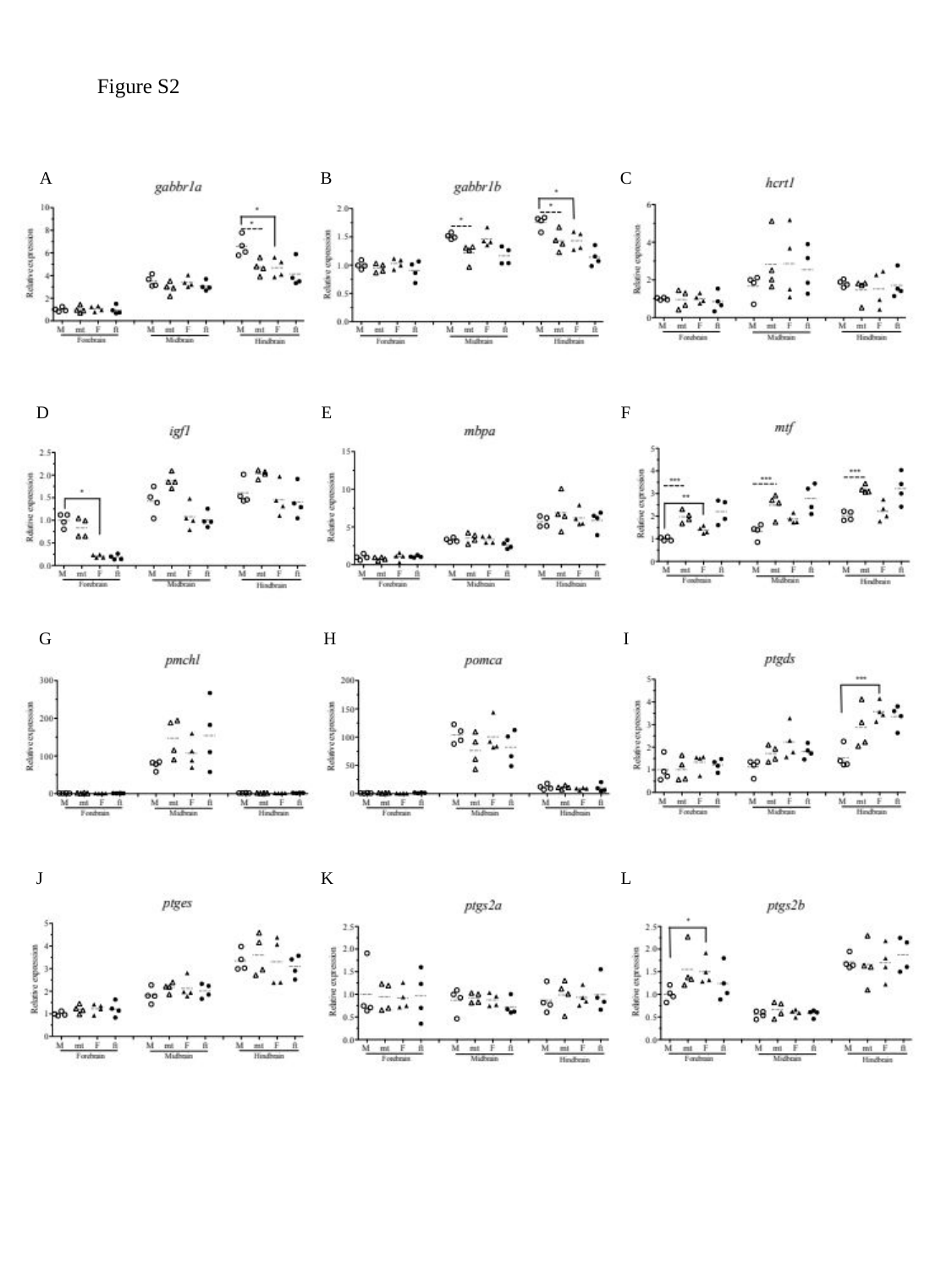

Figure S2
B
A
C
E
D
F
H
G
I
K
J
L

## Slide 3
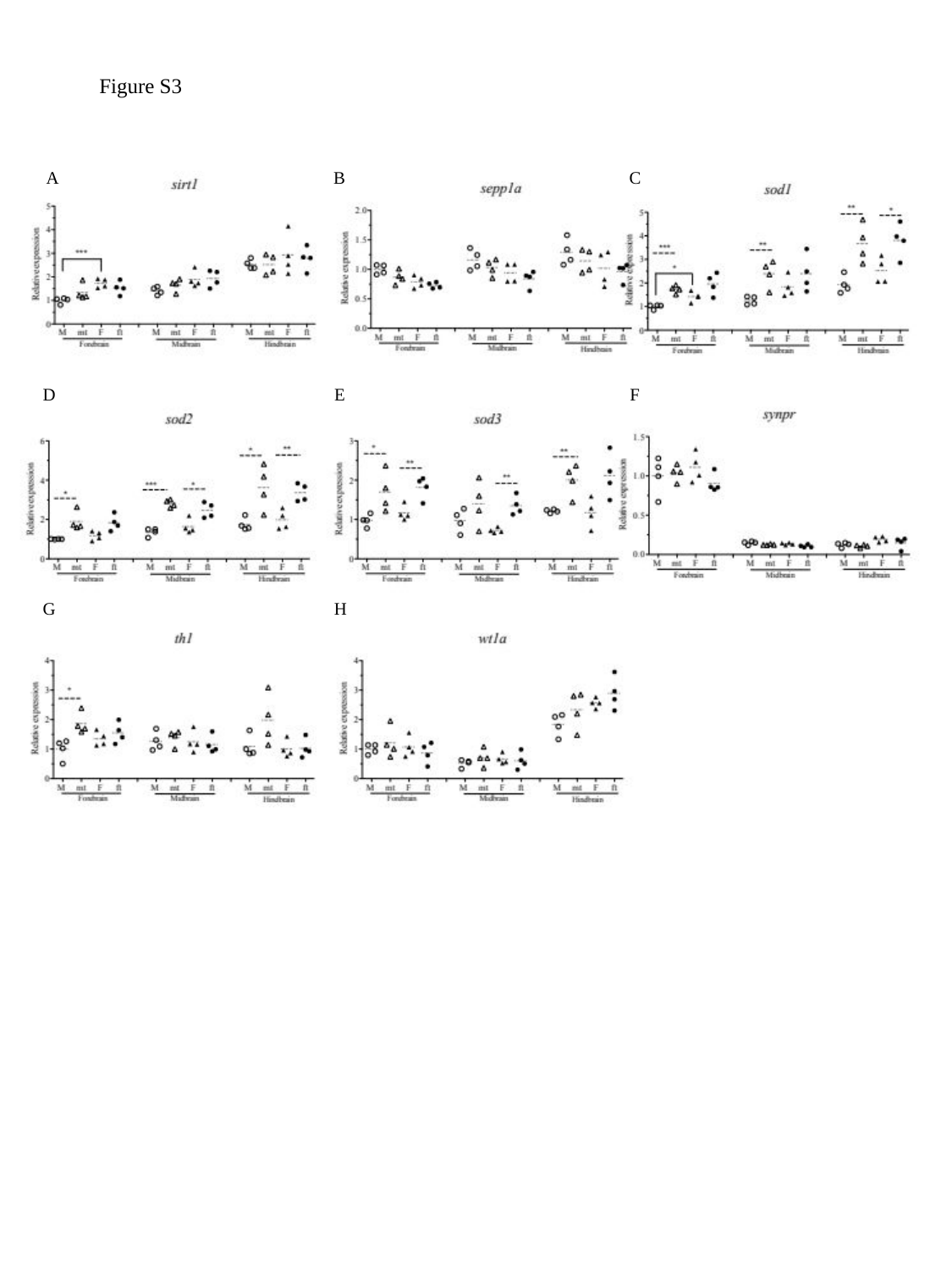

Figure S3
B
A
C
E
D
F
H
G

Supplement: Additional file 2: — Figure S1. Male and female zebrafish were exposed separately to 25 nM E2 for 24 h and the brain tissue was isolated and dissected into three regions. RNA was isolated from individual samples followed by cDNA synthesis and qRT-PCR analysis. Statistical significance was determined as outlined in the materials and methods section. Statistically significant differences were determined using Student’s t test (*p < 0.05; **p < 0.01; *** p < 0.001). n = 4. Figure S2. Male and female zebrafish were exposed separately to 25 nM E2 for 24 h and the brain tissue was isolated followed by cDNA synthesis and qRT-PCR analysis. Statistical significance was determined as outlined in the materials and methods section. Statistically significant differences were determined using Student’s t test (*p < 0.05; *** p < 0.001). n = 4. Figure S3. Male and female zebrafish were exposed separately to 25 nM E2 for 24 h and the brain tissue was isolated followed by cDNA synthesis and qRT-PCR analysis. Statistical significance was determined as outlined in the materials and methods section. Statistically significant differences were determined using Student’s t test (*p < 0.05; **p < 0.01; *** p < 0.001). n = 4. [file 12993_2015_68_MOESM2_ESM.pptx]
